# Investigation of Amino Acids As Herbicides for Control of *Orobanche minor* Parasitism in Red Clover

**DOI:** 10.3389/fpls.2017.00842

**Published:** 2017-05-22

**Authors:** Mónica Fernández-Aparicio, Alexandre Bernard, Laurent Falchetto, Pascal Marget, Bruno Chauvel, Christian Steinberg, Cindy E. Morris, Stephanie Gibot-Leclerc, Angela Boari, Maurizio Vurro, David A. Bohan, David C. Sands, Xavier Reboud

**Affiliations:** ^1^Agroécologie, AgroSup Dijon, INRA, Université Bourgogne Franche-ComtéDijon, France; ^2^CSIC, Institute for Sustainable AgricultureCórdoba, Spain; ^3^INRA, UE0115 Domaine Expérimental d’Epoisses,Bretenière, France; ^4^INRA, UR0407 Pathologie VégétaleMontfavet, France; ^5^CNR, Institute of Sciences of Food ProductionBari, Italy; ^6^Department of Plant Sciences & Plant Pathology, Montana State University, BozemanMT, United States

**Keywords:** parasitic weeds, methionine, alternatives for crop protection, animal feed supplements, germination, host attachment, herbicidal activity of amino acids, sustainable agriculture

## Abstract

Certain amino acids induce inhibitory effects in plant growth due to feedback inhibition of metabolic pathways. The inhibition patterns depend on plant species and the plant developmental stage. Those amino acids with inhibitory action on specific weeds could be utilized as herbicides, however, their use for weed control has not been put into practice. *Orobanche minor* is a weed that parasitizes red clover. *O. minor* germination is stimulated by clover root exudates. The subsequent seedling is an obligated parasite that must attach quickly to the clover root to withdraw its nutrients. Early development of *O. minor* is vulnerable to amino acid inhibition and therefore, a series of *in vitro*, rhizotron, and field experiments were conducted to investigate the potential of amino acids to inhibit *O. minor* parasitism. In *in vitro* experiments it was found that among a collection of 20 protein amino acids, lysine, methionine and tryptophan strongly interfere with *O. minor* early development. Field research confirmed their inhibitory effect but revealed that methionine was more effective than lysine and tryptophan, and that two successive methionine applications at 308 and 543 growing degree days inhibited *O. minor* emergence in red clover up to 67%. We investigated additional effects with potential to influence the practical use of amino acids against broomrape weeds, whether the herbicidal effect may be reversible by other amino acids exuded by host plants or may be amplified by inducing host resistance barriers against *O. minor* penetration. This paper suggests that amino acids may have the potential to be integrated into biorational programs of broomrape management.

## Introduction

Broomrape weeds (*Orobanche* and *Phelipanche* spp.) are angiosperms that lack photosynthetic competence and a root system. They thrive in agricultural ecosystems parasitizing a wide range of host crops in the Apiaceae, Asteraceae, Brassicaceae, Fabaceae, and Solanaceae (Parker and Riches, 1993). Among them, *Orobanche minor* Sm., is widely distributed inducing major economic damage in clover and lucerne crops (Lins et al., 2007; Parker, 2013). In eastern France, the main host crop is red clover (*Trifolium pratense* L.) in which, besides a strong reduction in clover harvest, *O. minor* seeds contaminate clover forage and seed yield resulting in a significant problem in crops for commercial seed production.

Broomrape seed germination is stimulated by host root exudates. Once germination is initiated, an infective radicle arises from the seed coat that grows to make contact with the host plant. Upon host root contact, a haustorium is induced at the tip of the broomrape radicle, with functions of host root invasion, vascular connection and nutrient withdrawal (Vaucher, 1823; Kuijt, 1969; Joel, 2013). Once the resource sink is established by the young parasite, the parasite initiates storage of host-derived nutrients in an underground parasitic organ called tubercle. This underground stage of parasitic development produces a strong injury in the crop (Eizenberg et al., 2005). Near the end of parasitic life cycle, underground shoots are developed in the tubercles that quickly emerge through the soil surface for flowering and seed set. Each flowering stalk can set up more than half million dust-like seeds easily dispersed by wind, farm machinery and crop seeds and therefore the build-up of parasitic seed bank is very efficient.

The control of broomrape weeds remains an elusive task in agriculture (Rubiales et al., 2009). Early diagnosis of broomrape infection is difficult due to the mostly underground growing habit of this weed. In addition, the close physical and chemical associations established by broomrape with the parasitized crop difficult the selective control of the weed without injuring the crop. In absence of host crop stimulation to germinate, broomrape seeds can last viable in agricultural soils for several decades being their seed bank extremely persistent. Once germinated the broomrape seedling does not survive longer than few days without host-derived nutritive supply. Control strategies interfering with broomrape germination and radicle growth inhibit host infection and broomrape reproduction producing a decline in the parasitic seed bank while meeting short-term farming expectations of crop productivity (Fernández-Aparicio et al., 2016). Once the parasitic seedling has made contact with the host root surface, host resistance to parasite invasion either natural, genetically engineered or elicited by biotic- or abiotic-inducing agents leads to death of the invading parasite before it causes strong injury in the crop (Gonsior et al., 2004; Aly, 2007; Kusumoto et al., 2007). Once the parasite has established resource sink and the tubercle started to develop, the control of broomrape weeds can be achieved by using glyphosate and imidazolinone herbicides (Hershenhorn et al., 2009). Those herbicides inhibit enzymes that catalyze key steps in the biosynthesis of aromatic and branched-chain amino acid, respectively. For efficacious action on broomrape weeds, the herbicide is applied on the foliage of herbicide-tolerant crop cultivars and then translocated systemically in active form towards the underground parasite via the haustorium (Lins et al., 2005; Eizenberg et al., 2006). Despite broomrape parasitism can be inhibited very efficiently by cultivation of resistant crops to broomrape infection or application of systemic herbicides, parasite resistant- or herbicide tolerant-cultivars are not always available for many crop species.

In addition, due to increasing concern about overdependence on synthetic herbicides there is a great demand for novel approaches in weed management programs. As a green alternative for weed control, the enzymes targeted in weeds by glyphosate and imidazolinone herbicides, can also be inhibited by the exogenous addition of certain amino acids (Sands and Pilgeram, 2009). The rationale is based in the mode of enzymatic regulation in amino acid biosynthetic pathways. Amino acid biosynthesis is an energy expensive process, and it is regulated by feedback inhibition of key enzymes by some of the amino acid products of the pathways they regulate. This feedback inhibition leads in plant growth inhibition (Bright et al., 1978), and it can be counteracted by exogenous addition of certain related amino acids in the same biosynthetic pathway. In addition, amino acids from unrelated pathways can rescue the plant growth by giving general growth stimulus or reactivating the targeted enzyme in the case of end product of competitive pathways. Active amino acids for plant growth inhibition and plant growth rescue are dependent on the plant species, the plant developmental stage and concentration of application (Henke et al., 1974) and therefore, the development of this strategy for weed management systems requires the determination of which amino acids and concentrations are efficacious against the targeted weed species and whether the inhibition is reversible by other amino acids potentially present in the soil (Sands and Pilgeram, 2009). Properly implemented this strategy assumes low toxicity for plants other than the targeted weed and low persistence in the soil through microbial metabolization. In addition, because the targeted enzymes are absent in animals it is also assumed low toxicity in them. Although this approach has been reported effective for control of Canada thistle, red bromegrass, kudzu and cannabis, the direct application of amino acids as herbicides has not been put into field practice yet (Sands and Pilgeram, 2009).

Germination and radicle growth of the parasitic weeds *Phelipanche ramosa* and *Orobanche crenata* have been found to be inhibited by certain amino acids (Vurro et al., 2006; Fernández-Aparicio et al., 2013). Therefore, amino acids could have the potential to be implemented into broomrape management systems as herbicides. Given the obligated parasitic growth of these weeds, the implementation of this approach requires to consider additional factors which could influence the herbicidal treatment. The growth habit of broomrape before host attachment is restricted to the crop rhizosphere because its germination is stimulated by host root exudates. In addition to germination factors, root exudates of host crop species are a significant source of amino acids. Glycine, serine, and alanine are abundant in root exudates, but also concentrations of glutamic acid, arginine, tyrosine, glutamine, or valine have been described to be significant in crop root exudates (Lesuffleur et al., 2007). Therefore, for development of this approach, it should be determined which of those amino acids could have the potential to counteract the herbicidal effect of the broomrape-inhibitory amino acids in case they were deposited in the soil by clover roots.

Another amino acid effect against parasitic weeds could be through the potential elicitation of broomrape resistance in the crop. It has been found that certain amino acids such as methionine induce resistance in other pathosystems (Sarosh et al., 2005). Induction of resistance barriers in the crop roots to parasitic weed penetration could extend the amino acid protective effect against the parasitic weed to parasitic stages beyond its attachment. Broomrape infection is susceptible to be inhibited by other abiotic elicitors of resistance in the host crop (Gonsior et al., 2004; Kusumoto et al., 2007) but the potential of methionine to elicit crop resistance to broomrape parasitism has never been investigated before.

Given the predicted low persistence of amino acid treatment in the soil (Sands and Pilgeram, 2009), the herbicidal effect of amino acids would be maximized if the timing of application is based on the occurrence of susceptible parasitic life stages. Broomrape development depends on temperature (Eizenberg et al., 2005) and therefore, the relation between temperature and underground phenology should be recorded locally under field conditions in order to ease the targeting of vulnerable parasitic life stages by amino acid treatment.

This research conducted laboratory and field experiments to evaluate the effect of direct application of amino acid as herbicides for field control of *O. minor* parasitism in red clover. This paper reports (i) the influence of single amino acids and combination of amino acids on *O. minor* growth previous to clover attachment. To achieve this, amino acids were applied *in vitro* to seeds and germination rate and radicle length were measured; (ii) the indirect effects of methionine on growth of *O. minor* after its attachment to clover roots. This was achieved by treating clover with methionine and measuring its resistance to *O. minor* haustorium penetration; (iii) to study under open field conditions the inhibitory effect of amino acids on *O. minor* parasitism in clover. To achieve this the amino acid was applied by irrigation at clover post-emergence during the occurrence of amino acid-sensitive *O. minor* stages. This paper proposes that the amino acid herbicidal approach could have the potential to be integrated into sustainable programs of broomrape management.

## Materials and Methods

### *In Vitro* Experiments

#### *In Vitro* Identification of Amino Acids with Inhibitory Activity on *O. minor* Parasitism at Host Pre-attached Stages

Sensitivity of *O. minor* germination and radicle growth to alanine, arginine, aspartate, cysteine, glutamic acid, glutamine, glycine, histidine, homoserine, isoleucine, leucine, lysine, methionine, phenylalanine, proline, serine, threonine, tryptophan, tyrosine, and valine was assessed *in vitro*. The *O. minor* seeds used were obtained in 2014 from *O. minor* plants infesting red clover field at INRA Experimental Unit of Epoisses, Bretenières (France). Seeds were collected from mature, dry *O. minor* inflorescences using a 0.5 mm mesh-size sieve and stored dry in the dark at room temperature until use.

*In vitro* bioassays were conducted to study the amino acid inhibitory activity on *O. minor* germination and radicle growth. One-year old *O. minor* seeds were surface sterilized by immersion in 1.5% (w/v) NaOCl, and 0.02% (v/v) Tween 20, and sonication for 2 min, rinsed thoroughly with sterile distilled water and dried in a laminar air flow cabinet. Inhibitory activity was tested as reported previously (Fernández-Aparicio et al., 2013). For germination, *Orobanche* seeds require a warm stratification period called conditioning, followed by chemical stimulation with germination-inducing factors (Lechat et al., 2012). Approximately 100 seeds of *O. minor* were placed separately on each of 183 glass fiber filter paper (GFFP) disks of 9 mm-diameter moistened with 50 μL of sterile distilled water. *O. minor* seeds were conditioned in the dark at 22°C for 7 days. The germination stimulant GR24 (Johnson et al., 1976) was used to promote germination of conditioned seeds. Test solutions of each amino acid were prepared in GR24 solution at three decreasing concentrations of the amino acid (5, 2.5, and 1.25 mM) but keeping constant the GR24 concentration (10^-6^M) in order to allow comparisons between treatments. GR24 solution (10^-6^M) without amino acid was used as control. GFFP disks containing the conditioned *O. minor* seeds were transferred inside laminar flow cabinet to sterile sheet of filter paper to remove excess of moisture and transferred to 10 cm-diameter Petri dishes. Aliquots of 50 μl of each test solution were applied to triplicated GFFP disks containing the *O. minor* conditioned seeds. Petri dishes were sealed with Parafilm and stored in the dark at 22°C to promote germination and radicle growth. Seven days later, *O. minor* seeds were observed under stereoscopic microscope at which time germination percentage was assessed by scoring the number of of seeds with and emerged radicle from 100 seeds in each of three replicate GFFP disks and radicle length was determined in 15 randomly selected germinated seeds in each of the three replicate GFFP disks. Amino acid-mediated reductions of radicle growth were expressed as a percent of the untreated control (GR24).

#### Inhibitory Action of Amino Acids on *O. minor* Parasitism at Host Post-attached Stages

We tested the hypothesis that amino acids could elicit a mechanism of clover resistance to *O. minor* penetration. We make this hypothesis based on the facts that certain amino acids such as methionine induce resistance in other pathosystems (Sarosh et al., 2005) and that abiotic elicitors of induced resistance have been shown effective to inhibit broomrape parasitism (Gonsior et al., 2004; Kusumoto et al., 2007). The tetraploid red clover cultivar Trevvio (RAGT semences) was chosen due to its high susceptibility to *O. minor* parasitism. Seeds of Trevvio were treated with 20 mM concentration of L-methionine (Sigma-Aldrich, St. Louis, MO, United States) at 22°C for 9 h. Then, imbibed clover seeds were placed in pots with perlite to allow clover germination and seedling growth during 10 days prior being transplanted to the minirhizotron system described by Fernández-Aparicio et al. (2008). One clover seedling developed from L-methionine imbibed seeds were transplanted per square Petri dishes filled with sterile perlite and covered in the upper surface by a sheet of GFFP. Clover seeds imbibed with distilled water (control treatment) were cultivated under the same conditions for comparisons. *O. minor* seeds collected, sterilized and conditioned as described in Section “*In Vitro* Identification of Amino Acids with Inhibitory Activity on *O. minor* Parasitism at Host Pre-attached Stages”, were treated with the germination stimulant GR24 and distributed at a proximate density of 50 seeds per cm^2^ on the GFFP on which the clover plants were developing their roots. After 27 days of *O. minor* inoculation, parasitic seedlings that made contact with clover roots were inspected under a stereoscopic microscope and classified as either (i) *O. minor* radicles attached to clover root surface which died as a consequence of unsuccessful penetration and vascular connection showing hypersensitive-like response at the attachment point, (ii) *O. minor* radicle that penetrated the host root and formed a healthy tubercle as consequence of successful nutrient transfer, (iii) *O. minor* radicles that penetrated the host root and formed tubercle that quickly turned necrotic and later died as a consequence of host-parasite late incompatible interaction, and (iv) “spider” stage, i.e., tubercles with rapid and healthy development as seen by initiation of crown roots. The effects of the methionine imbibition treatment on *O. minor* infection was determined by calculation of the following four parameters: infection success (percent of contacted *O. minor* radicles that successfully invaded the host root), hypersensitive-like response (percent of contacted *O. minor* radicles that died before vascular connection showing browning area at the host attachment point), necrosis development in tubercle (percent of total formed tubercles that died at early stages of development becoming necrotic) and ‘spider’ development in tubercle (percent of formed tubercles that initiated crown roots as consequence of successful parasitism).

#### *In Vitro* Validation of Inhibitory Activity of Amino Acids Formulated as Supplements in Animal Feed

Among the group of *O. minor*-inhibitory amino acids identified in Section “*In vitro* Identification of Amino Acids with Inhibitory Activity on *O. minor* Parasitism at Host Pre-attached Stages”, tryptophan and the aspartate-derived amino acids: lysine, methionine and threonine were selected as candidates for field control. This choice was on the basis of the preliminary results and took into account the ready availability of these amino acids at large commercial scale as animal feed supplements. Prior to field research, the inhibitory activity of these amino acids was validated in the form of lysine-, threonine- and tryptophan-based animal feed formulations (Ajinomoto Heartland, Inc, Japan). In addition, methionine hydroxyl analog with 100% equivalency to DL-methionine (Novus International, St. Charles, IL, United States) was tested in two different formulations: formulation 1: Alimet^®^; and formulation 2: non-commercialized experimental formulation. Activity of each formulation was tested at three decreasing concentrations of the amino acid (10, 5, and 2.5 mM) on *O. minor* seed germination and radicle growth as described in Section “*In Vitro* Identification of Amino Acids with Inhibitory Activity on *O. minor* Parasitism at Host Pre-attached Stages”.

#### Determination Whether Inhibitory Action of Candidate Amino Acids for Field Control is Reversed by Amino Acids Commonly Exuded by Crop Roots

*O. minor* seeds and seedlings placed in the soil within the potentially host infective distance are exposed to crop root exudates which may contain significant amounts of amino acids (Lesuffleur et al., 2007). To assess if herbicidal effects of lysine, methionine and tryptophan could be reversible by other amino acids, their respective inhibitory activity was studied mixed in pairs with the amino acid collection tested in Section “*In Vitro* Identification of Amino Acids with Inhibitory Activity on *O. minor* Parasitism at Host Pre-attached Stages”. Rescue of 2.5 mM lysine-, methionine-, and tryptophan-toxicity was assessed by adding in pairs the other 19 amino acids at 2.5 mM concentration and the joint activity of each mixture on *O. minor* germination and radicle growth compared with the inhibitory action performed by their respective control: single solutions of lysine, methionine, or tryptophan at 2.5 mM. Bioassay and data collection were performed as described in Section “*In Vitro* Identification of Amino Acids with Inhibitory Activity on *O. minor* Parasitism at Host Pre-attached Stages”.

### Field Validation of Amino Acid Inhibitory Activity on *O. minor* Parasitism

Field experimentation was performed at INRA Experimental Unit of Epoisses, Bretenières, France (5°05’56”E; 47°14’20”N) with long history of approximately uniform seed bank infestation level of *O. minor* specialized in red clover. The soil at the experimental site (35 first cm layer) has a silt clay texture (sand 6.4%, silt 50.6%, clay 43%), with pH 7.97, 2.5% organic matter, C/N ratio of 10.5 and a Cation Exchange Capacity of 29.6 cmol(+) kg^-1^ soil. The red clover cultivar Trevvio was chosen due to its high susceptibility to *O. minor* parasitism. On April 7, 2015, 84 plots (3 m× 1.5 m) were mechanically sown with Trevvio seeds at a density of 37.5 kg/ha. In addition, in order to ensure dense and uniform *O. minor* seed bank, the sowing machine (Plotseed, Wintersteiger equipped with “Accord CX” disk sowing equipment) distributed 0.5 ml (approximately 63000) *O. minor* seeds per plot.

**FIGURE 1:**
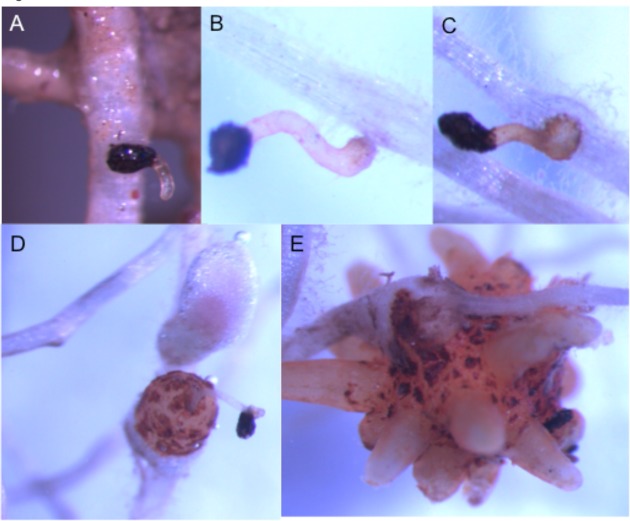
***Orobanche minor* seedlings at successive stages of the infection of clover roots observed during a field trial in Burgundy, France. (A)** *O. minor* germination. **(B)** *O. minor* adhesion to host root. **(C)** Host penetration. **(D)** Vascular connection. **(E)** Tubercle development with initiation of ‘spider’ stage.

The timing of amino acid treatment was determined by inspecting the *O. minor* underground development in control plots. Starting at red clover sowing, soil and air temperature was recorded every hour with temperature data loggers, respectively, placed at 5 cm below the soil surface and 1 m above soil surface in control plots. Approximately ten clover plants were randomly sampled every 3–4 days and brought to lab. Roots were submerged in water allowing the excess of soil gently separate from the roots but maintaining the rhizosheath intact as much as possible. Then, clover roots were inspected under stereoscopic microscope. Dates were recorded for each of the following *O. minor* phenological stages: germination, radicle adhesion to host root surface, radicle penetration, vascular connection, tubercle development, development of “spider” stage (**Figure 1**). For each date, temperatures were converted to growing degree day (GDD) accumulated from clover sowing date considering base temperature as 0°C (McMaster and Wilhelm, 1997; Eizenberg et al., 2005). The timing of amino acid treatment was chosen according with observations of *O. minor* underground phenology. Two amino acid treatments were applied at 22 and 36 days after clover sowing. They, respectively, corresponded to clover-induced *O. minor* germination (282 GDD air/ 308 GDD soil) and initiation of *O. minor* radicle penetration into clover root (494 GDD air/543 GDD soil). For each application date, 10 treatments were included as follows. (A) Four animal feed formulations described in Section “*In Vitro* Validation of Inhibitory Activity of Amino Acids Formulated as Supplements in Animal Feed”: methionine hydroxyl analog formulations 1 and 2 (Novus International, St. Charles, IL, United States ), lysine and tryptophan (Ajinomoto Heartland, Inc, Japan) treatments at 10 and 20 mM concentrations. (B) L-methionine (Sigma) treatment at 20 mM concentration, and (C) water (control treatment). For each treatment, the corresponding amino acids formulation were mixed with water at the described concentrations and used to irrigate the plots at a rate of 6 L m^-2^ of amino acid solution. For each treatment, the test solution was prepared based on the amino acid content in each formulation: L-methionine (100%), methionine hydroxyl analog formulation 1 (88%), methionine hydroxyl analogue formulation 2 (95%), lysine (50%), tryptophan (98%). The experimental design was a randomized block with four replications. Each treated plot including the control-plot was surrounded by two untreated plots at their immediate right and left side. Plots were managed under traditional culture. Weeds other than *O. minor* were carefully removed by hand.

The level of *O. minor* parasitism on each plot was assessed counting the number of emerged *O. minor* plants per plot. In order to avoid the error due to unavoidable heterogeneous distribution of seeds across the trial, this parameter was standardized as ‘*O. minor* emergence referred to the control’ calculated as percentage to the mean value of emerged broomrapes per respective control plots located at right and left of each test plots.

For statistical analysis, percentages were transformed to arcsine square roots (transformed value = 180/Π x arcsine [√(%/100)]) to normalize data and stabilize variance throughout the data range and subjected to analysis of variance using SAS 9.4 (SAS Institute Inc., Cary, NC, United States). The significance of mean differences between each treatment against the control was evaluated by the two-sided Dunnett test. The null hypothesis was rejected at the level of 0.05.

## Results

### Sensitivity of *O. minor* Germination and Radicle Growth to Exogenous Addition of Amino Acids

**FIGURE 2:**
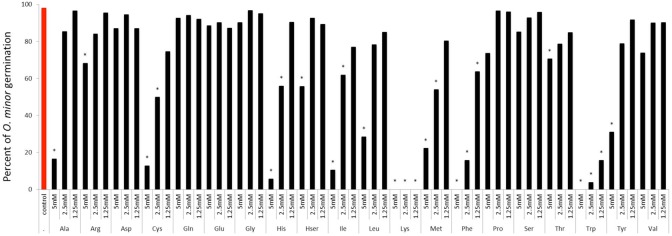
**Influence of amino acid treatments on *O. minor in vitro* germination.** Treatment solutions of alanine, arginine, aspartate, cysteine, glutamic acid, glutamine, glycine, histidine, homoserine, isoleucine, leucine, lysine, methionine, phenylalanine, proline, serine, threonine, tryptophan, tyrosine, and valine along with a germination stimulant GR24 were applied to *O. minor* seeds at a range of 5–1.25 mM and their inhibitory activity in germination rated 7 days later as percentage of germination in each of three replicated disks. Analysis of variance was applied to transformed replicate data. ^∗^Indicates differences of each treatment compared with the control GR24 assessed by Dunnett’s test at the 0.05 level.

The first experiment was conducted to identify which amino acids inhibit *O. minor* host-preattached stages. Twenty amino acids in pure form were individually applied to seeds of *O. minor* in combination with the germination stimulant GR24 and levels of germination and radicle growth inhibition rated in comparison with the control (GR24 with no amino acid). The sensitivity of *O. minor* germination to amino acids is shown in **Figure 2**. Arginine, aspartic acid, glutamic acid, glutamine, glycine, homoserine, proline, serine, threonine, and valine caused low or non-significant reduction in percent seed germination in comparison with that of the control. The magnitude of reduction in germination was greater in alanine, cysteine, histidine, isoleucine, leucine, methionine and tyrosine which significantly inhibited *O. minor* germination at concentration of 5 mM although lower concentrations of these amino acids induced low or negligible effect. Lastly, the greatest inhibitory activity was identified in lysine, phenylalanine, and tryptophan that completely inhibited *O. minor* germination at all concentrations tested.

**FIGURE 3:**
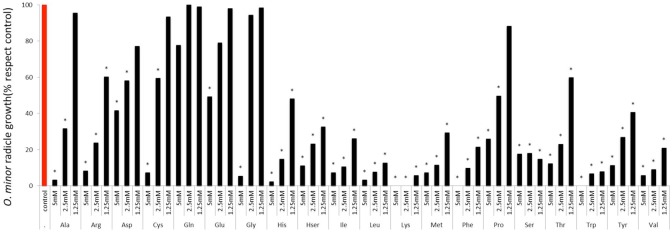
**Influence of amino acid treatments on *O. minor* radicle growth *in vitro*.** Treatment solutions of alanine, arginine, aspartate, cysteine, glutamic acid, glutamine, glycine, histidine, homoserine, isoleucine, leucine, lysine, methionine, phenylalanine, proline, serine, threonine, tryptophan, tyrosine, and valine along with a germination stimulant GR24 were applied to *O. minor* seeds at a range of 5–1.25 mM and their activity in growth reduction rated 7 days later in each of three replicated disks and expressed as a percent of the untreated control activity. Analysis of variance was applied to transformed replicate data. ^∗^Indicates differences of each treatment compared with the control assessed by Dunnett’s test at the 0.05 level.

The sensitivity of *O. minor* radicle growth to exogenous amino acids is shown in **Figure 3**. Except for glutamine, all amino acids inhibited radicle growth at 5 mM and therefore this concentration did not allow discrimination between amino acids for their inhibitory action. At 1.25 mM, the lowest concentration tested, it was possible to identify strong inhibition activity in histidine, homoserine, isoleucine, leucine, lysine, methionine, phenylalanine, serine, tryptophan, tyrosine, and valine. Although little effect was induced at 1.25 mM by alanine, arginine, proline, and threonine, they caused more than 50% radicle growth inhibition at concentration of 2.5 mM and higher. A group of low or non-inhibitory amino acids of radicle growth was formed by aspartic acid, cysteine, glutamic acid, glutamine, and glycine, which induced less than 50% radicle growth inhibition at concentration range of 2.5–1.25 mM.

### *In Vitro* Efficacy of Amino Acid Supplements for Animal Feed as Readily Deployable Formulations for Field Application

**FIGURE 4:**
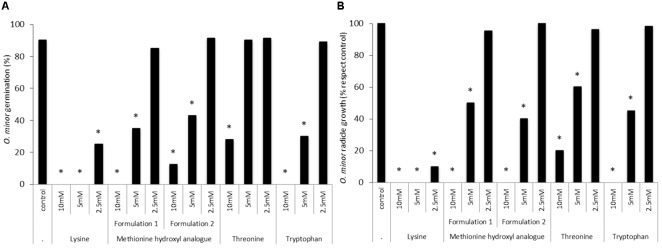
**Effects of lysine-, methionine hydroxyl analog-, threonine-, and tryptophan-based feed supplements in *O. minor in vitro* development. (A)** Inhibition of *O. minor* germination. **(B)** Inhibition of *O. minor* radicle growth. ^∗^Indicates differences of each treatment compared with the control (2.5 mM methionine treatment) assessed by Dunnett’s test at the 0.05 level.

*In vitro* tests were used to validate the herbicidal activity of methionine, lysine, threonine and tryptophan in the form of animal feed-based formulation. **Figure 4** shows that lysine-based formulation induced the highest inhibition of *O. minor* germination and radicle growth at all concentration tested being this strong herbicidal activity in agreement with activity found by lysine in the pure form (**Figures 2, 3**). **Figure 4** also shows that the activity of methionine- and tryptophan-based feed supplements was slightly lower than that observed when tested in pure form. *O. minor* development was inhibited by their commercial formulations at 10 and 5 mM but not at the 2.5 mM concentration which was inhibitory when those amino acids were tested in pure form. Threonine-based animal feed supplements displayed the lowest inhibitory activity being only active at the highest concentration tested and therefore it was not included for further tests.

### Potential Reversible Effects of Amino Acid Herbicidal Activity by Other Amino Acids

**FIGURE 5:**
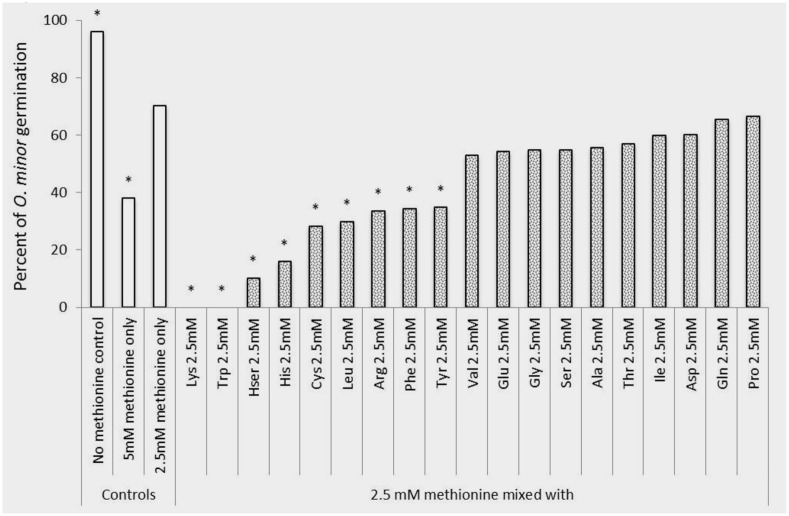
**Rescue of methionine inhibition of *O. minor* germination induced by application of 2.5mM of alanine, arginine, aspartate, cysteine, glutamic acid, glutamine, glycine, histidine, homoserine, isoleucine, leucine, lysine, methionine, phenylalanine, proline, serine, threonine, tryptophan, tyrosine, and valine.** ^∗^Indicates differences of each treatment compared with the control (germination observed in 2.5 mM methionine treatment) assessed by Dunnett’s test at the 0.05 level.

**FIGURE 6:**
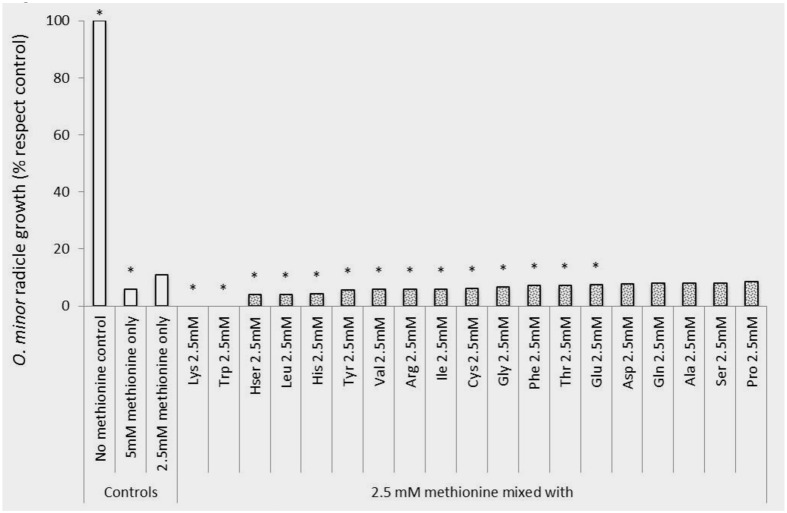
**Rescue of methionine inhibition of *O. minor* radicle growth induced by application of 2.5mM alanine, arginine, aspartate, cysteine, glutamic acid, glutamine, glycine, histidine, homoserine, isoleucine, leucine, lysine, methionine, phenylalanine, proline, serine, threonine, tryptophan, tyrosine, and valine in *in vitro* assays.** ^∗^ Indicates differences of each treatment compared with the control (radicle growth observed in 2.5 mM methionine treatment) assessed by Dunnett’s test at the 0.05 level.

An *in vitro* experiment was conducted to verify that the herbicidal activity identified in lysine, methionine, and tryptophan could not be reversed by other amino acids potentially present in the crop rhizosphere. **Figures 5, 6** show that methionine inhibition of *O. minor* germination and radicle growth was not reversed by any other amino acid tested resulting the amino acid mixtures in equal or higher *O. minor* inhibition levels when compared to their respective methionine control solutions. The lysine or tryptophan control solutions induced complete inhibition of *O. minor* germination and radicle growth and this herbicidal activity was not counteracted by addition of any other amino acid (data not shown).

### Timing of Amino Acid Application

Amino acid inhibition of weed parasitism targets early parasitism stages, i.e., germination and host attachment. The kinetics of clover infection by *O. minor* in relation with temperature under field conditions in Burgundy (France) was determined by regular inspections of clover root system from clover sowing. **Table 1** shows dates transformed to soil and air GDD for *O. minor* germination, radicle adhesion to clover root surface, host penetration, vascular connection, tubercle formation, full spider stage development (**Figure 1**). In addition to characterization of temperature-related underground development of *O. minor* in real farming conditions in Burgundy, these measurements allowed us to determine an adequate timing for amino acid treatments at 282 GDD air/308 GDD soil (corresponding to clover-induced *O. minor* germination) and 494 GDD air/543 GDD soil (corresponding to initiation of *O. minor* radicle penetration into clover root).

**Table 1:** Growing degree-days (GDD) accumulated since clover sowing at each *O. minor* parasitism stage observed based on measurements of soil temperature (5 cm below the soil surface) and air temperature (1 m above soil surface).

		*O. minor* parasitism stages						
Growing degree days (GDD) (C)	*Clover emergence*	Germination	Radicle adhesion	Host penetration	Vascular connection established	Tubercle development	Spider stage	Shoot meristem
	
	April 15, 2015	April 29, 2015	May 5, 2015	May 13, 2015	May 19, 2015	May 28, 2015	June 2, 2015	June 9, 2015
GDD Soil	110.2	307.5	412.9	542.6	654.1	797.3	886.9	1035.6
GDD Air	100.1	281.9	385.2	493.5	581.7	703.7	788.0	938.3

### Effect of Amino Acid Field Treatments in the Parasitism of *O. minor* in Red Clover

**FIGURE 7:**
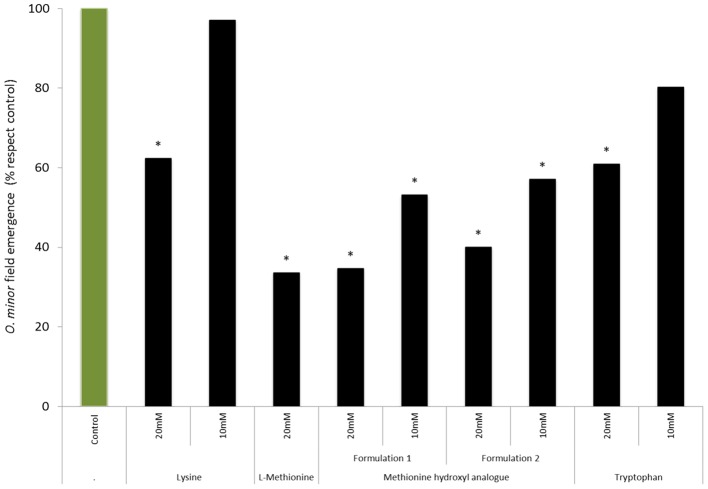
**Influence of lysine, L-methionine, methionine hydroxyl analog, and tryptophan field treatments in the emergence of *O. minor* in red clover during a field trial in Burgundy, France in 2014.** ^∗^Indicates differences of each treatment compared with the control assessed by Dunnett’s test at the 0.05 level.

A field experiment in Burgundy was conducted to study the effects of amino acid treatments (lysine, methionine, methionine hydroxyl analog, and tryptophan) at concentration (20 and 10 mM) on *O. minor* field emergence (**Figure 7**). In control-plots, an average of 21 *O. minor* plants emerged per square meter of soil surface. The stronger herbicidal activity observed *in vitro* by lysine and tryptophan relative to that induced by methionine was not validated in the field. Lysine- and tryptophan-based treatments at 10 mM did not reduce *O. minor* emergence in field. Increasing the concentration of lysine and tryptophan up to 20 mM lead, respectively, to 37 and 39% reductions of *O. minor* emergence with respect to the *O. minor* emergence observed in the control. By contrast, methionine-based formulations were more effective in reducing *O. minor* field parasitism. Methionine at 20 mM either as L-methionine pure form or as methionine hydroxyl analog (formulations 1 and 2) respectively, reduced by 67, 65, and 60% the emergence of *O. minor* in comparison with the control. The inhibitory activity was maintained over 40% in methionine hydroxyl analog formulations when applied at 10 mM. None of the tested amino acid formulations were inhibitory to clover plants.

### Induction of Clover Resistance: A Potential Enhancement of Herbicidal Efficacy

We studied the possibility that methionine could inhibit the invasion of *O. minor* haustorium into clover roots by inducing a resistant reaction in clover. **Table 2** shows the results of microscope observation of *O. minor* infection on roots of methionine-treated clover plants. Despite methionine-treated clover plants did not show significant symptoms of hypersensitive-like response at the host-parasite interface, the *O. minor* infection success m as the percentage of successful host-parasite vascular connections out of total parasitic radicles that made contact with host roots was slightly but significantly reduced in methionine-treated clover plants in comparison with control clover plants. The parasitic development beyond vascular connection measured as the percentage of vascular connections that developed into spider stage was not affected by methionine treatment.

**Table 2:** Protective effect of methionine imbibition of red clover seeds against *O. minor* parasitism.

Clover seed imbibition treatment	Total number of *O. minor* tubercles per clover plant	Infection success (%)	Hypersensitive-like response (%)	Necrosis of tubercle (%)	‘Spider’ development (%)
Control	28.0	44.0	0.4	0.0	28.6
20mM L-methionine	17.4^∗^	26.3^∗^	1.5^ns^	0.0	29.3^ns^

## Discussion

Herbicidal activity of amino acids has been studied previously being the amino acid delivered either by root exudation of allelopathic plants (Bertin et al., 2007) or by over-excreting pathogens (Nzioki et al., 2016). Under laboratory conditions, exogenous supply of amino acids to tomato rhizosphere inhibited *Phelipanche* parasitism (Vurro et al., 2006). The present work is consistent with those reports certifying the value of amino acids for weed control by studying for the first time the direct field delivery of amino acids as a management strategy against *O. minor* parasitism in red clover.

We have investigated some of the main mechanisms potentially involved in the weed inhibition activity. Previously, the germination sensitivity of the parasitic plant *P. ramosa* to amino acid inhibition was described *in vitro* (Vurro et al., 2006). We tested the hypothesis that amino acid inhibition and effective doses vary depending on the broomrape species targeted. In *in vitro* assays, *O. minor* germination was strongly inhibited by lysine, phenylalanine and tryptophan but not affected by any other amino acid at the concentration range tested except when assayed at the highest concentration at which *O. minor* seeds showed sensitivity to alanine, cysteine, histidine, isoleucine, leucine, methionine and tyrosine. Although the effective doses differ, our results are in agreement with those reported for *P. ramosa* by Vurro et al. (2006) in that lysine, methionine, alanine, and histidine have the potential to inhibit broomrape germination. In contrast, *O. minor* sensitivity differs in that several amino acids such as proline and glycine, reported to be strong inhibitors of *P. ramosa* (Vurro et al., 2006), do not inhibit *O. minor* germination certifying that patterns of germination inhibition triggered by exogenous amino acids differ between broomrape species.

Although radicle growth inhibition by amino acids have been reported before (Vurro et al., 2006; Fernández-Aparicio et al., 2013), sensitivity of broomrape radicle growth to exogenous amino acids have not been systematically studied before the present work. Our *in vitro* screening demonstrated that the process of radicle growth is more sensitive than germination to amino acid inhibitory activity. In fact, every amino acid tested except for glutamine, glutamic acid and aspartic acid, promoted general levels of radicle growth inhibition at the highest concentration tested. Applying each amino acid at reduced concentrations allowed us to identify strong inhibitors, many of them also being active as inhibitors of germination, i.e., lysine, phenylalanine, and tryptophan but also alanine, isoleucine, histidine, leucine, methionine, and tyrosine. This indicates that the inhibition phenomenon could be affecting common metabolic pathways required for germination and radicle growth processes. Amino acids that differentially targeted either the processes of germination or radicle growth were identified less frequently. Among them serine, threonine and valine inhibited *O. minor* radicle growth but did not inhibit its germination at the concentration tested while cysteine inhibited germination but its activity in radicle growth was only observed at high concentrations in which general inhibition was observed.

Although the direct delivery of phytoinhibitory amino acids as alternative to synthetic herbicides is a promising weed control strategy, it lacks field optimization (Sands and Pilgeram, 2009). Among the *in vitro*-identified top inhibitory amino acids, the commercial availability at a large scale of lysine-, methionine-, and tryptophan-based supplements for animal feed enabled us to select those amino acids as candidates for field research. Lysine and methionine are commonly called aspartate-derived amino acids produced from aspartate in plants via a branched pathway (Jander and Joshi, 2010). Tryptophan is an aromatic amino acid synthesized via the shikimate pathway followed by the branched aromatic amino acid metabolic pathway (Tzin and Galili, 2010).

For broomrape control, the inhibitory amino acid needs to be delivered in the crop rhizosphere, the narrow region of soil in which broomrape seed germination is triggered by crop root exudates and the infectious radicle invades the crop root. Crop root exudates are also a source of amino acids (Lesuffleur et al., 2007) which could reverse the herbicidal effect due to complex regulation events (Bonner and Jensen, 1997; Curien et al., 2005; Jander and Joshi, 2010). Therefore, the broomrape vulnerability observed *in vitro* to amino acid inhibition might have little consequences in the natural environment if the herbicidal amino acid co-exists in the rhizosphere with another amino acid that abolishes the growth inhibition. Our *in vitro* results suggest that the *O. minor* inhibition caused by lysine, methionine, and tryptophan is not reversed by amino acids frequently exuded by roots of clover and other crops.

The *in vitro* screening identified lysine and tryptophan as strong inhibitors of *O. minor* germination and radicle growth. Those biological processes are crucial in the life cycle of obligated parasitic weeds enabling host root recognition and invasion. The germination inhibitory activity of methionine was low, however, it strongly reduces the maximum length of the infective radicle which also may lead to inhibition of broomrape parasitism by inhibiting the capacity of the parasite to reach and therefore invade the host (Vurro et al., 2006; Fernández-Aparicio et al., 2013). Because amino acid treatments target those highly coordinated but ephemeral parasitic stages the timing of application is instrumental for the success of this technique especially in light of the fact that the persistence of amino acids in agricultural soils is predicted to be low due to microbial metabolization (Sands and Pilgeram, 2009). Development of broomrape life cycle is highly synchronized with host phenology and for each broomrape-crop association, temperature is the main factor affecting such synchronization (Eizenberg et al., 2005). GDD-based models for *O. minor* field development from tubercle formation have been efficient in assisting the decision of application timing of systemic herbicides (Eizenberg et al., 2005; Lins et al., 2005). However, our amino acid treatment targets earlier stages in *Orobanche* parasitism and therefore in order to standardize adequate timing of amino acid field applications, we monitored in the field from clover sowing the early underground stages of *O. minor* parasitism. From the field screening we determined that two applications of methionine hydroxyl analog at the parasitic stages of germination and host invasion suppressed *O. minor* parasitism. The suppressive effect of methionine hydroxyl analog in field was better than lysine and tryptophan formulations despite the fact that lysine and tryptophan were better inhibitors of *O. minor in vitro* development. Although faster microbial metabolization could influence the lower effect of lysine and tryptophan relative to methionine, we considered the possibility that besides the direct effect of methionine in *O. minor* preattached seedling, an additional mechanism could be protecting the crop through a methionine-elicited mechanism of induced resistance against *O. minor* penetration. In fact, methionine has been described to elicit defense related enzymes and resistance in other crop-pathogen associations (Sarosh et al., 2005). In order to dissect the effect of methionine as potential elicitor of clover resistance against *O. minor* from the methionine direct inhibitory effect in *O. minor* germination and radicle growth we imbibed clover seeds with methionine and cultivated the subsequent seedlings in rhizotrons in absence of methionine. A hypersensitive response was not observed at the *O. minor*-clover interface. However, despite no difference being observed in the development of *O. minor* pre-attached seedlings relative to the control, the number of established parasites relative to the number of parasitic seedlings that made contact with the host root was significantly reduced in methionine-protected clover plants. This study provides justification for molecular studies to unveil the precise cause of the infection inhibition by methionine and offers the possibility to refine the application conditions of amino acids such as methionine-coated clover seeds.

## Author Contributions

MF-A designed, implemented the studies, collected and analyzed the data and wrote the manuscript. ABe, LF, PM, BC, CS, CM, XR contributed in field design and implementation. ABe, LF, PM, SG-L, ABo, MV, DB, DS, XR provided materials and laboratory equipment. BC, CS, CM, MV, DS, XR reviewed and discussed the final version of the manuscript.

## Conflict of Interest Statement

The authors declare that the research was conducted in the absence of any commercial or financial relationships that could be construed as a potential conflict of interest.
